# EEG Frequency Tagging Reveals the Integration of Form and Motion Cues into the Perception of Group Movement

**DOI:** 10.1093/cercor/bhab385

**Published:** 2021-11-04

**Authors:** Emiel Cracco, Haeeun Lee, Goedele van Belle, Lisa Quenon, Patrick Haggard, Bruno Rossion, Guido Orgs

**Affiliations:** Department of Experimental Psychology, Ghent University, 9000 Ghent, Belgium; Department of Psychology, Goldsmiths, University of London, SE14 6NW London, UK; Psychological Sciences Research Institute, Université Catholique de Louvain, 1340 Ottignies-Louvain-la-Neuve, Belgium; Institute of Neuroscience, Université Catholique de Louvain, 1000 Brussels, Belgium; Institute of Cognitive Neuroscience, University College London, WC1N 3AZ London, UK; Université de Lorraine, CNRS, CRAN, F-54000 Nancy, France; CHRU-Nancy, Service de Neurologie, F-54000 Nancy, France; Department of Psychology, Goldsmiths, University of London, SE14 6NW London, UK

**Keywords:** biological motion perception, frequency tagging, groups, perceptual binding, synchrony

## Abstract

The human brain has dedicated mechanisms for processing other people’s movements. Previous research has revealed how these mechanisms contribute to perceiving the movements of individuals but has left open how we perceive groups of people moving together. Across three experiments, we test whether movement perception depends on the spatiotemporal relationships among the movements of multiple agents. In Experiment 1, we combine EEG frequency tagging with apparent human motion and show that posture and movement perception can be dissociated at harmonically related frequencies of stimulus presentation. We then show that movement but not posture processing is enhanced when observing multiple agents move in synchrony. Movement processing was strongest for fluently moving synchronous groups (Experiment 2) and was perturbed by inversion (Experiment 3). Our findings suggest that processing group movement relies on binding body postures into movements and individual movements into groups. Enhanced perceptual processing of movement synchrony may form the basis for higher order social phenomena such as group alignment and its social consequences.

## Introduction

Much of human behavior occurs in group (Wiltermuth and Heath 2009; Shamay-Tsoory et al. 2019). As a result, to correctly interpret social situations, we often have to process multiple people moving together. Watching people coordinate their movements is known to activate reward-related brain areas (Eskenazi et al. 2015). It also signals affiliation (Lakens and Stel 2011; Marques-Quinteiro et al. 2019; Wilson and Gos 2019) and adds to the aesthetic appreciation of both music (Hagen and Bryant 2003) and dance (Vicary et al. 2017). Surprisingly, however, only very little is known about how coordinated group behavior is processed in the brain. Indeed, existing models of biological motion perception have largely focused on processing the movements of individuals (Giese and Poggio 2003) or dyads (Hovaidi-Ardestani et al. 2018). These models have proposed two pathways for processing biological motion. In the *structure-from-motion* pathway, perception of biological motion arises from an analysis of the kinematics of observed movements; in the *motion-from-structure* pathway, it instead arises from combining sequences of static body snapshots into fluent motion (Giese and Poggio 2003; Lange and Lappe 2006). This dual pathway structure is supported by evidence that biological motion perception does not require moving stimuli but can also be induced by sequences of static body images (Orgs and Haggard 2011; Orgs et al. 2011, 2013). Such apparent movement perception relies on neurons that integrate static body information over time (Jellema and Perrett 2003; Singer and Sheinberg 2010), in extra-striate visual and motor areas of the brain (Downing et al. 2006; Stevens et al. 2006; Orgs et al. 2016).

Importantly, although both motion-from-structure and structure-from-motion processing typically involve human stimuli, they are by no means specific to human movement (Giese and Poggio 2003; Lange and Lappe 2006). For example, perceptual learning studies have shown that the mechanisms used to process human movements are also used to process movements made by complex artificial shapes (Jastorff et al. 2006, 2009). In both cases, movement processing involves both a local part-based and a global configural route (Jastorff et al. 2006; Urgesi et al. 2007). Crucially, however, the configural route can be disrupted by stimulus inversion (Maurer et al. 2002) and both body (Reed et al. 2003) and movement perception (Grossman and Blake 2001; Orgs et al. 2011) display such inversion effects. As a result, biological motion processing can be viewed as a special case of configural motion processing.

Configural motion processing is essential for recognizing and interpreting other people’s behavior (Giese and Poggio 2003). But how does it scale up to groups? Recent studies indicate that the brain can simultaneously represent the actions of multiple agents (Cracco et al. 2015, 2016, 2019; Cracco and Brass 2018a, 2018b, 2018c; Cracco and Cooper 2019). However, whether multiple agents are perceived as a group is determined not by the number of agents but by the relationships between their movements (Ip et al. 2006). Here, we test the hypothesis that movement perception is sensitive to synchrony between movements. Synchrony defines what we perceive as a group (Braddick et al. 2001; Wagemans et al. 2012), purely based on sustained temporal coupling of movement trajectories (Brick and Boker 2011). It is also an important social cue that signals group cohesion (Lakens and Stel 2011; Marques-Quinteiro et al. 2019; Wilson and Gos 2019). Hence, we hypothesized that the brain would bind synchronous movements into a single group movement to which it would respond more strongly.

**Figure 1:**
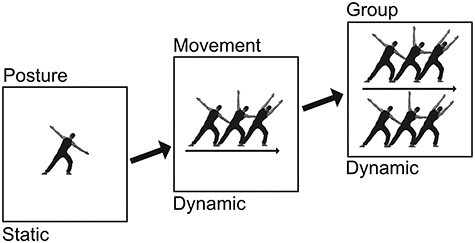
Three stages of configural movement processing. The first stage is posture perception, in which static postures are processed individually (Downing et al. 2006). The second stage is movement perception, in which fluent sequences of body postures are integrated over time into a continuous movement percept (Orgs et al. 2011, 2013, 2016). Finally, the third stage is group perception, in which the movements of the individual agents are bound into groups based on synchrony (Wagemans et al. 2012). Stages that are specific to configural stimulus shapes should be disrupted by inversion. This has previously been shown for posture (Reed et al. 2003) and movement (Orgs et al. 2011) perception, but whether it also applies to group perception is an open question. Note that body movements are depicted by showing three consecutive postures of the performed movement.

Specifically, we tested the hypothesis that the perception of group movement in the motion-from-structure pathway proceeds along three stages (see Fig. 1). In the first two stages, the movements of the individual agents are processed separately. In stage 1, static body postures are processed (Downing et al. 2006). In stage 2, these body postures are then integrated over time into a continuous movement percept (Giese and Poggio 2003; Lange and Lappe 2006). As explained above, these first two stages are sensitive to body configuration (Reed et al. 2003; Orgs et al. 2011). Finally, in stage 3, the relations between the individual movements are analyzed and interrelated movements are bound together into a single group movement based on the principles of synchrony and common fate (Wagemans et al. 2012). Whether this third stage is also sensitive to body configuration is an open question.

**Figure 2:**
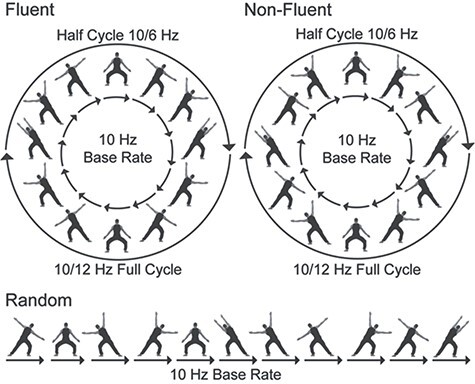
Stimulus sequences for the fluent, non-fluent, and random conditions of Experiment 1. Images were presented at a base rate of 10 Hz. In the fluent condition, images were ordered to induce a coherent movement percept. This percept was perturbed in the non-fluent and random conditions. In the non-fluent condition, the movement percept was perturbed by reordering the images to achieve maximum visual displacement between successive body postures. In the random condition, it was perturbed by presenting the images at random. Fluent and non-fluent sequences had the same symmetrical structure, with the second half of the sequence mirroring the first half played backwards. Hence, in these sequences, a turning point occurred at a frequency of 10/6 Hz (half cycle) and the full sequence repeated at a frequency of 10/12 Hz (full cycle). In the fluent condition, the half cycle point coincides with movement completion. As a result, half cycle responses should primarily capture dynamic movement and group perception (Stage 2 and 3). The full cycle point instead coincides with the completion of the full posture sequence and full cycle responses should therefore primarily capture static posture processing (Stage 1).

To test our hypothesis, we developed a new EEG paradigm that combines apparent biological motion (Orgs et al. 2011, 2013) with frequency tagging (Norcia et al. 2015) by generating cyclical sequences of 12 body postures that produced either fluent or non-fluent apparent motion. Importantly, these sequences were symmetrical, with the second half of each sequence mirroring the first half played backwards (Supplementary Videos; Fig. 2). According to the logic of frequency tagging (Norcia et al. 2015), this should result in brain responses at three different frequencies: a response coupled to individual image presentation, at base rate (BR), a response coupled to the turning point in the sequence, at half cycle rate (BR/6), and a response coupled to the completion of the entire image sequence, at full cycle rate (BR/12).

In Experiment 1, we tested whether our approach could capture the integration of postures into movements for a single agent only (Giese and Poggio 2003; Lange and Lappe 2006). To this end, we measured brain activity elicited by fluent, non-fluent, and random sequences. Fluent sequences consisted of body postures in their natural order. This elicited an apparent motion percept that was perturbed in the non-fluent sequences by reordering the postures in a non-fluent order and in the random sequences by presenting the images randomly (Orgs et al. 2013, 2016). Although all three sequences were built from the same postures, they differed in their temporal structure. Random sequences lacked temporal structure. Fluent and non-fluent sequences, on the other hand, both had the same symmetrical structure, but this structure became salient only in fluent sequences. This is because the primary percept in fluent sequences is a series of movements, presented here at half cycle rate (Shiffrar and Freyd 1990; Orgs and Haggard 2011; Orgs et al. 2011, 2013, 2016), whereas in non-fluent sequences, it is a series of body postures, presented here at full cycle rate (Downing et al. 2006; Orgs et al. 2013, 2016). As a result, if our task captures the temporal binding of bodies into movements, half cycle responses should be stronger for fluent than for non-fluent sequences, whereas full cycle responses should be stronger for non-fluent than for fluent sequences.

In Experiments 2–3, we then tested how the brain distinguishes independent from group movements. Specifically, we tested the hypothesis that it does so by processing temporally related movements not as multiple individual movements but as a single group movement (Wagemans et al. 2012). If true, then neural responses at the frequency of apparent movement (half cycle) should be more pronounced for synchronous than for asynchronous observed movements (see also Alp et al. 2017 for a different approach to study motion synchrony with frequency tagging). If group perception builds on movement perception, this synchrony effect should further be perturbed by temporal scrambling (Experiment 2), and if it is specific to configural shapes, it should additionally be perturbed by stimulus inversion (Experiment 3).

### Open Science Statement

The data and analysis scripts of all three experiments are available on the Open Science Framework via the following link: https://osf.io/bs8n7/.

## Experiment 1

### Methods

#### Participants

Ten healthy volunteers with normal or corrected-to-normal vision participated in the experiment (9 female, *M*_age_ = 22.78, SD_age_ = 2.44, range_age_ = 19–26, 1 unknown sex and age). Although a sample size of *N* = 10 allows us to detect only large effects *d*_z_ ≥ 0.72 (Lakens et al. 2018), such effects are to be expected considering that both apparent biological motion perception (e.g., Shiffrar and Freyd 1990; Stevens et al. 2006; Orgs et al. 2011) and the influence of temporal scrambling on biological motion perception (Downing et al. 2006; Lange and Lappe 2007) are reliably observed even with small samples (Smith and Little 2018), and considering the high signal-to-noise ratio of EEG frequency tagging (Regan 1966; Norcia et al. 2015). All participants signed an informed consent before the experiment and were paid 10 Euros in exchange for their participation. Experiment 1 was conducted at the Université Catholique Louvain and was approved by the local ethics committee.

#### Task, Stimuli, and Procedure

The experiment was programmed in MatLab 2009 using Psychtoolbox (Brainard 1997). In the experiment, participants saw repeating sequences of 12 gray-scale body images (12 × 12°) presented on a gray background (Fig. 2). There were two experimental conditions (fluent and non-fluent) and one control condition (random). In the random sequences, images were presented randomly. In contrast, the fluent and non-fluent sequences both had a fixed, symmetrical structure in which the second half of the sequence mirrored the first half played backwards. In fluent sequences, images were arranged to form a rhythmical dance movement representing a dancer moving from left to right and back from right to left. In the non-fluent condition, these same 12 images were rearranged into a sequence with maximum visual displacement between successive body postures. As a result, even though both sequences were symmetrical, the symmetry was salient only in fluent sequences.

The above stimulation procedure should produce responses at three different frequencies that correspond to distinct features of the image sequence (Fig. 2). First, presenting images at a fixed pace should produce a response at the stimulation frequency of 10 Hz (base rate). This stimulation frequency was based on previous behavioral research showing that a 10-Hz presentation rate produces a reliable percept of apparent motion (Orgs et al. 2011). Second, in the fluent and non-fluent sequences only, two additional responses should be produced every sixth (half cycle, at 1.67 Hz) and twelfth image (full cycle, at 0.83 Hz). These responses are coupled, respectively, to the symmetrical turning point in the sequence and to the point at which the full image sequence repeats. Crucially, this means that half and full cycle responses do not reflect the processing of specific images, but rather the processing of an event coupled to those images, namely the turning point (half cycle) and repetition (full cycle) of the sequence.

Fluent sequences are known to be perceived as a series of movements (Orgs and Haggard 2011; Orgs et al. 2011, 2013, 2016) and non-fluent sequences as a series of independent body postures (Downing et al. 2006; Orgs et al. 2013, 2016). As movement completion occurs at half cycle rate in our task, fluent sequences should make the half cycle point more salient and hence should primarily elicit responses at half cycle frequencies. In contrast, posture sequences repeat at full cycle rate. Non-fluent sequences should therefore make the full cycle point more salient and should primarily elicit responses at full cycle frequencies. Stated differently, half cycle responses should primarily reflect dynamic visual processing (i.e., movements), whereas full cycle responses should primarily reflect static visual processing (i.e., body postures). Moreover, in the random condition, where neither posture sequences nor movements repeat, both half- and full cycle responses should be absent.

The three conditions were presented blockwise in randomized order, with five blocks per condition. Each block consisted of a 120-s video with a 10-s fade in and 10-s fade out. The fluent and non-fluent videos were created by repeating the corresponding 12-image sequence 100 times and the random videos by presenting a random 1200-image sequence (Supplementary Video 1–3). To maintain attention, participants were instructed to fixate on a gray cross in the center of the screen and to press the space bar each time its color changed briefly (200 ms) to red (Rossion et al. 2012).

#### E‌EG Recording and Preprocessing

EEG was recorded from 128 Ag/AgCl active electrodes using a Biosemi EEG system and a sampling rate of 512 Hz. Vertical and horizontal eye movement were measured using four additional electrodes placed on the outer canthus of each eye and in the inferior and superior areas of the right orbit. Electrodes were referenced online to AFz, and their impedances were kept below 10 kΩ. All EEG data were offline processed using Letswave 6 (https://www.letswave.org/). Raw data were band-pass filtered using a fourth-order Butterworth filter with cut-off values of 0.1–100 Hz and segmented according to the experimental conditions (−2 to 122 s). Next, eye movement artifacts were removed by applying ICA on the merged segmented data. Specifically, we analyzed the first 10 components and removed one component for blinks and one or two components related to eye movements. The average number of eye blinks per block ranged from 20 to 22 for the three conditions. There were no significant differences between conditions, although the difference between the random and non-fluent condition approached significance (*P* = 0.057, other *Ps* ≥ 0.310). After ICA, faulty or excessively noisy electrodes (<1% on average) were interpolated using the data from the three closest neighboring electrodes. The signal was then re-referenced with respect to the average of all electrodes, before cropping the segments into 96 s epochs (12–108 s). At 10 Hz, this ensures that all relevant harmonics are multiples of the epoch duration and therefore that target frequencies are captured by a single frequency bin, which increases the signal-to-noise ratio. Finally, the trials within each condition were averaged and a Fast Fourier Transform (FFT) was applied to transform the data of each electrode to normalized (divided by N/2) amplitudes (μV) in the frequency domain (from 0 to 256 Hz).

#### E‌EG Analysis

Frequency tagging does not only elicit responses at the tagged frequencies, but also at harmonics of those frequencies. For each of the three expected responses, at base rate (10 Hz), full cycle (0.83 Hz), and half cycle (1.67 Hz), we extracted the first 10 significant harmonics up until 100 Hz. To determine significance, we pooled all 128 electrodes of the grand-averaged signal, computed *z*-scores comparing each frequency bin to its 20 surrounding (except directly adjacent) bins, and selected the first 10 harmonics with *z* > 2.32 (i.e., *P*_one-tailed_ < 0.01; Retter and Rossion, 2016). Importantly, the three frequencies are harmonically related. Therefore, to minimize overlap, the full cycle response was calculated using only those harmonics that did not overlap with the half cycle harmonics (i.e., the odd harmonics) and the half cycle response was calculated using only those harmonics that did not overlap with the base rate harmonics. Accordingly, the full cycle response was calculated as the sum of amplitudes at 0.83, 2.50, 4.17, 5.83, 7.50, 9.17, 10.83, 12.50, 14.17, and 15.83 Hz. The half cycle response was calculated as the sum of amplitudes at 1.67, 3.33, 5.00, 6.67, 8.33, 11.67, 13.33, 15.00, 16.67, and 18.33 Hz. Finally, the base rate response was calculated as the sum of 8 instead of 10 harmonics, 10, 20, 30, 40, 60, 70, 80, and 90 Hz, excluding bins capturing electrical noise at 50 and 100 Hz. Amplitudes were baseline-corrected by subtracting the signal from the 20 surrounding (except directly adjacent) bins from each frequency bin (signal-to-noise subtraction; SNS). Because baseline-subtracted amplitudes were used, the summed response in the absence of signal is expected to be 0 (Retter and Rossion 2016).

To prevent selection bias, the electrodes entered into the analysis were chosen by averaging the topographies of each response across participants and conditions (Luck and Gaspelin 2017). This revealed four clusters: a middle posterior cluster with a maximum at Oz, two lateral posterior clusters with maxima at PO7 and PO8, and a frontocentral cluster with a maximum at FCz (Supplementary Fig. 1). To keep cluster size identical across regions of interest, the response in each cluster was quantified by taking five electrodes centered around the maximum electrode.

The resulting data for each response were analyzed in R with a condition (fluent, non-fluent, or random) × region (left posterior, middle posterior, right posterior, or middle central) repeated measures ANOVA. ANOVA degrees of freedom were corrected for violation of sphericity using the Greenhouse–Geisser correction whenever Mauchly’s sphericity test was significant (*P* < 0.05). Unless otherwise specified, all tests are two tailed. All pairwise tests are accompanied by Bayes Factors (BFs) that quantify the evidence for the tested effects. See Supplementary Table 1 for means and standard deviations per condition.

### Results

**Figure 3:**
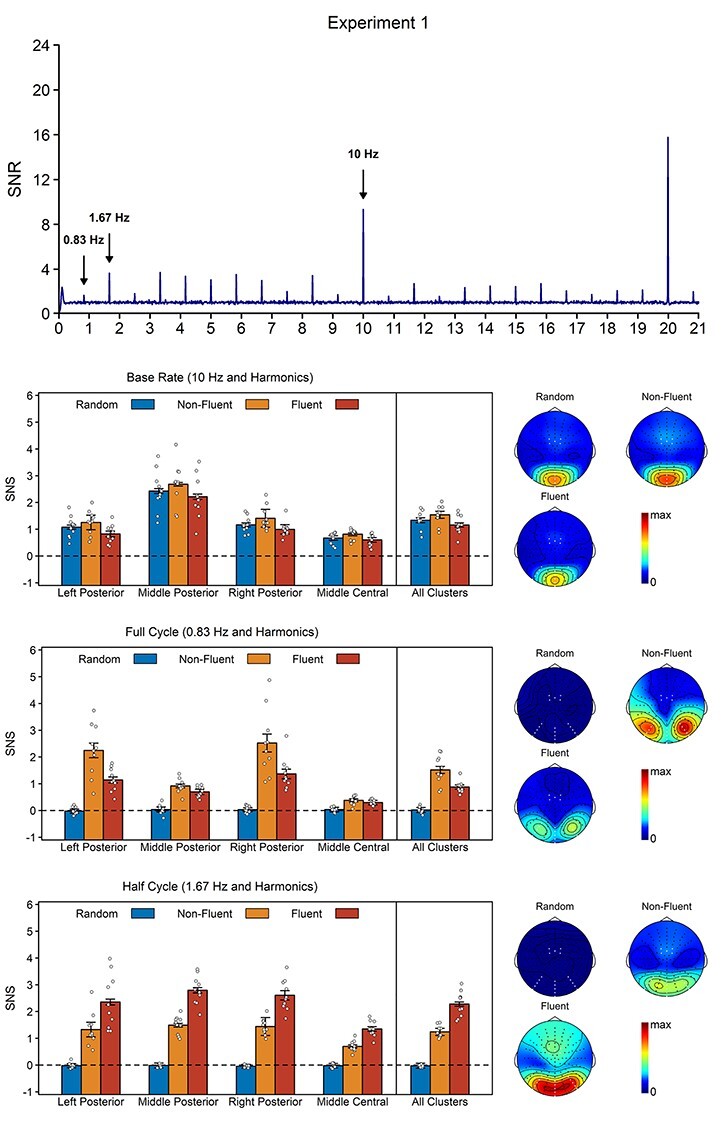
Results of Experiment 1. Top. Signal-to-noise ratio (SNR) across all participants, conditions, and electrodes of interest. See Supplementary Fig. 3 for plots per condition. Bottom. Noise-subtracted amplitudes (signal-noise-subtraction; SNS) per condition and response, together with their topographies. Topographies are scaled from 0 to the maximum amplitude across conditions. The white dots on the topographies indicate the electrodes included in the analysis. The white dots on the barplots represent the data of the individual participants. Error bars are standard errors of the mean (SEMs) corrected for within-subject designs (Morey 2008).

Base rate is coupled to image presentation. As a result, base rate responses should capture a-specific processing of the images, regardless of posture. If this is the case, then these responses should be strongest in the non-fluent condition, where the visual change from image to image is largest, and, crucially, should also be visible in the random condition, where neither postures nor movements repeat predictably. In line with this hypothesis, the base rate ANOVA revealed a main effect of condition, *F*(2, 18) = 25.23, *P* < 0.001, η*_p_*^2^ = 0.74, with stronger responses in the non-fluent condition than in both the fluent, *t*(9) = 5.46, *P* < 0.001, BF_10_ = 87.98, *d*_z_ = 1.73, and random condition, *t*(9) = 4.26, *P* = 0.002, BF_10_ = 21.86, *d*_z_ = 1.35, and with stronger responses in the random condition than in the fluent condition, *t*(9) = 4.61, *P* = 0.001, BF_10_ = 33.32, *d*_z_ = 1.46. Thus, the base rate response was strongest in the non-fluent condition but was also present in the random condition, *t*(9) = 12.63, *P*_one-tailed_ < 0.001, BF_10_ = 5.69 × 10^4^, *d*_z_ = 4.00. Topographic maps revealed a focal, middle posterior topography (Fig. 3). This was supported by a main effect of region, *F*(1.58, 14.25) = 42.57, *P* < 0.001, η*_p_*^2^ = 0.83, indicating that the base rate response was stronger in the middle posterior cluster than in the two lateral posterior clusters, all *t*(9) ≥ 5.21, *P* < 0.001, BF_10_ ≥ 66.31, *d*_z_ ≥ 1.65, and stronger in the three posterior clusters than in the central cluster, all *t*(9) ≥ 3.73, *P* ≤ 0.005, BF_10_ ≥ 11.37, *d*_z_ ≥ 1.18.

Full cycle rate is coupled to the completion of the full sequence and full cycle responses should therefore capture the processing of specific posture sequences. If true, these responses should be strongest in the non-fluent condition, where the primary percept is a repeating posture sequence. However, unlike base rate responses, full cycle responses should not be visible in the random condition, as there is no consistent repetition of the sequences there. In line with this hypothesis, the full cycle ANOVA revealed a main effect of condition, *F*(2, 18) = 52.36, *P* < 0.001, η*_p_*^2^ = 0.85, with stronger responses in the non-fluent than in the fluent condition, *t*(9) = 3.97, *P* = 0.003, BF_10_ = 15.31, *d*_z_ = 1.25, and stronger responses in both the fluent and non-fluent conditions than in the random condition, both *t*(9) ≥ 8.53, *P* < 0.001, BF_10_ ≥ 1.62 × 10^3^, *d*_z_ ≥ 2.70. As predicted, full cycle responses in the random condition did not differ from baseline, *t*(9) = 0.57, *P*_one-tailed_ = 0.293, BF_10_ = 0.49, *d*_z_ = 0.18. In contrast to base rate responses, topographic maps indicated that the full cycle response was characterized by a lateralized posterior topography (Fig. 3). This was supported by a main effect of region, *F*(2.03, 18.23) = 24.88, *P* < 0.001, η*_p_*^2^ = 0.73, showing that full cycle responses were stronger in the two lateral posterior clusters than in the other clusters, all *t*(9) ≥ 4.36, *P* ≤ 0.002, BF_10_ ≥ 24.72, *d*_z_ ≥ 1.38, and by a condition × region interaction, *F*(2.60, 23.43) = 20.03, *P* < 0.001, η*_p_*^2^ = 0.69, showing that the fluency effect (fluent vs. non-fluent) was significant in the two lateral clusters, t(9) ≥ 3.59, p ≤ 0.006, BF_10_ ≥ 9.61, *d*_z_ ≥ 1.14, but not in the two other clusters, *t*(9) ≤ 2.09, *P* ≥ 0.066, BF_10_ ≤ 1.44, *d*_z_ ≤ 0.66.

Finally, half cycle rate is coupled to the symmetrical turning point of the sequence. Importantly, this turning point is present in both fluent and non-fluent sequences, but is coupled to a salient change of apparent movement direction only in fluent sequences. Stated differently, in the fluent condition, the primary percept is no longer a sequence of postures occurring at full cycle but a sequence of movements occurring at half cycle. As a result, if half cycle responses capture the binding of postures into movements, they should be strongest in the fluent condition. Moreover, they should again be absent in the random condition. Supporting this hypothesis, the half cycle analysis revealed a main effect of condition, *F*(2, 18) = 169.53, *P* < 0.001, η*_p_*^2^ = 0.95, with stronger responses in the fluent than in the non-fluent condition, *t*(9) = 7.51, *P* < 0.001, BF_10_ = 678.64, *d*_z_ = 2.38, and stronger responses in both the fluent and non-fluent condition than in the random condition, both *t*(9) ≥ 15.56, *P* < 0.001, BF_10_ ≥ 1.39 × 10^5^, *d*_z_ ≥ 4.92. Responses in the random condition did not differ from baseline, *t*(9) = −1.89, *P*_one-tailed_ = 0.954, BF_10_ = 0.13, *d*_z_ = −0.60. Topographic maps revealed that the half cycle topography included not only a posterior cluster, but also a second weaker cluster over frontocentral electrodes (Fig. 3). This was supported by a main effect of region, *F*(1.53, 13.77) = 13.82, *P* = 0.001, η*_p_*^2^ = 0.61, showing that half cycle responses were stronger in the posterior clusters than in the central cluster, all *t*(9) ≥ 3.46, *P* ≤ 0.007, BF_10_ ≥ 8.14, *d*_z_ ≥ 1.10, and by a condition × region interaction, *F*(6, 54) = 11.83, *P* < 0.001, η*_p_*^2^ = 0.57, showing that the effect of fluency (fluent vs non-fluent) was larger in the middle, *t*(9) = 5.17, *P* < 0.001, BF_10_ = 63.75, *d*_z_ = 1.64, and right posterior cluster, *t*(9) = 4.88, *P* < 0.001, BF_10_ = 45.57, *d*_z_ = 1.54, and to a lesser extent in the left posterior cluster, *t*(9) = 2.11, *P* = 0.065, BF_10_ = 1.46, *d*_z_ = 0.67, than in the central cluster, even though it was significant in all clusters, all *t*(9) ≥ 5.06, *P* < 0.001, BF_10_ ≥ 56.24, *d*_z_ ≥ 1.60.

### Interim Discussion

Experiment 1 sought to validate EEG frequency tagging as a tool to dissociate static (Stage 1) and dynamic components (Stage 2) of apparent movement perception (see Fig. 1). To this end, we presented repeating sequences of 12 body images (full cycle) completing a movement every sixth image (half cycle). The results revealed that half cycle responses were strongest when the images were ordered to produce a fluent movement percept, whereas full cycle and base rate responses were strongest when the images were ordered to preclude such a percept (Orgs and Haggard 2011; Orgs et al. 2011, 2013, 2016). Thus, neural responses at half cycle *increased* with the perceptual saliency of apparent movement but decreased with the saliency of static body images, while neural responses at full cycle and base rate decreased with the perceptual saliency of apparent movement but increased with the saliency of static body images.

In addition to being influenced differently by our experimental manipulations, base rate, full cycle, and half cycle responses also had distinct topographies. Although it is difficult to relate these topographies to specific brain regions, the obtained activation clusters are consistent with the hypothesis of a processing hierarchy. More specifically, they suggest a hierarchy in which base rate responses reflect a-specific image processing in early visual areas, full cycle responses reflect static processing of specific postures in lateralized, higher-order visual areas, and half cycle responses reflect dynamic movement perception in visual as well as frontocentral areas, potentially indicating motor involvement (Arnstein et al. 2011).

In sum, Experiment 1 shows that we could capture the perceptual binding of successive postures into a continuous movement percept (Stages 1 and 2 of Fig. 1) at harmonically related but dissociable frequencies. Yet, the main question of this study remains to be addressed: How does the brain distinguish multiple agents moving independently from multiple agents moving together? Experiments 2 and 3 test the hypothesis that this relies on a third stage of configural movement perception that uses perceptual grouping principles such as synchrony (Wagemans et al. 2012) to bind multiple individuals’ movements into collective group movement. Experiment 1 showed that half cycle responses captured movement perception. Therefore, if temporally related movements are bound together into group representations, half cycle responses should be sensitive to whether observed movements align synchronously across the different agents. Experiments 2 and 3 test this hypothesis. In addition, Experiment 2 also tests whether binding postures into movements is a prerequisite for binding movements into groups. In other words, it tests if movement grouping indeed forms a third stage in the motion-from-structure pathway. If so, then it should require intact movement representations and the synchrony effect should be stronger for fluent than for non-fluent sequences. Finally, Experiment 3 tests if this movement grouping process is specific to movements of familiar, configural shapes, by testing whether it is stronger for upright than for inverted bodies.

## Experiments 2–3

### Methods

#### Participants

Two fully independent samples of 20 healthy volunteers participated in Experiment 2 (16 female, *M*_age_ = 26.85, SD_age_ = 6.47, range_age_ = 20–50) and Experiment 3 (14 female, *M*_age_ = 25.80, SD_age_ = 7.10, range_age_ = 18–49). Sample sizes were determined by an a-priori power analysis indicating that 19 participants were necessary to obtain 90% power to detect effect sizes one-third the size of the half cycle fluency effect in Experiment 1. The power analysis was based on the fact that interactions can reasonably be expected to be between 1/2th and 1/4th the size of the main effects they influence (Giner-Sorolla 2018). In both experiments, one participant had to be excluded because large artifacts across the scalp and throughout the entire experiment made the data uninterpretable. Therefore, the final sample in both experiments comprised 19 participants. All participants signed an informed consent prior to the experiment and were paid £20 in exchange for their participation. Both experiments were conducted at Goldsmiths College, University of London and were approved by the local ethics committee.

#### Task, Stimuli, and Procedure

Both experiments were programmed in PsychoPy (Peirce et al. 2019). The overall procedure was similar to Experiment 1, except that participants now saw not one but four agents, organized in a square grid around the fixation cross, performing fluent or non-fluent movements (Experiment 2), in upright or inverted orientation (Experiment 3), either in or out of synchrony (Figs 5 and 6). The angular size of each agent was 6.59 × 6.59 and the angular size of the entire stimulus display was 13.18 × 13.18. Movement fluency was manipulated in the same way as in Experiment 1 and synchrony was manipulated by making the agents start from the same (synchrony) or different (asynchrony) positions in the sequence (Wilson and Gos 2019). The four starting positions in the asynchronous condition were chosen to maximize perceived asynchrony as judged by the researchers and were the same for all participants. However, which agent started from which position was counterbalanced across participants. Note that we did not include non-fluent sequences in Experiment 3 to avoid that the effect of body inversion would be masked by the more salient manipulation of temporal scrambling and to not make the experiment excessively long.

Experiments 2–3 used a different presentation rate than Experiment 1. That is, instead of presenting images at a rate of 10 Hz, we now used a presentation rate of 7.5 Hz. This was done because a slower presentation rate made the asynchronous condition appear less synchronous. In line with Experiment 1, all conditions were presented blockwise in randomized order, with five blocks per condition. However, in contrast to Experiment 1, we now used videos of 128 s instead of videos of 120 s and used an 8 s fade in and fade out period. Videos were presented on a white background and were created by repeating the relevant 12-image sequence 80 times (Supplementary Videos 4 and 5). To maintain attention and minimize eye movements, participants were asked to focus on a black fixation cross in the center of the screen and to press the space bar each time its color changed briefly (267 ms) to red (Rossion et al. 2012). Before the experiment proper, participants completed one practice block where the body postures of all four agents were presented randomly, similar to the random condition of Experiment 1. Finally, after the experiment, participants did a brief rating task where they saw a shortened (25 s) video of each condition and were asked to rate the synchrony and complexity of the video as well as how much they liked it, on a scale from 0 to 100.

#### E‌EG Recording and Preprocessing

EEG was recorded from 64 Ag/AgCl active electrodes using a Biosemi EEG system and a sampling rate of 512 Hz. Vertical and horizontal eye movement were measured using four additional electrodes placed on the outer canthus of each eye and in the inferior and superior areas of the left orbit. All electrodes were online referenced to the left and right ear lobes during recording and their impedances were kept below 10 kΩ. All EEG data was offline processed using Letswave 6 (https://www.letswave.org/). Raw data was band-pass filtered using a fourth-order Butterworth filter with cut-off values of 0.1–100 Hz and segmented according to the experimental conditions (−2 to 130 s). Next, eye movement artifacts were removed by applying ICA on the merged segmented data, using the same approach as in Experiment 1. The average number of eye blinks per block in Experiment 2 ranged from 20 to 21 for the four conditions, with no significant effects of synchrony, fluency, or their interaction (all *P* ≥ 0.431). The average number of eye blinks per block in Experiment 3 ranged from 23 to 24 for the four conditions, with no significant effects of synchrony, configuration, or their interaction (all *P* ≥ 0.321). After ICA, faulty or excessively noisy electrodes (<1% on average) were interpolated using data from the three closest neighboring electrodes. In addition, in Experiment 2, a complete block was discarded for a single participant because a large artifact across the scalp disproportionally biased the signal. The signal was then re-referenced with respect to the average of all electrodes, before cropping the segments into 112 s epochs (8–120 s). At 7.5 Hz, this ensures that all relevant harmonics are multiples of the epoch duration. Finally, the trials within each condition were averaged and an FFT was applied to transform the data of each electrode to normalized (divided by *N*/2) amplitudes (μV) in the frequency domain (from 0 to 256 Hz).

#### Data Analysis

Synchrony, complexity, and liking ratings were analyzed with synchrony (synchronous or asynchronous) × fluency (fluent or non-fluent) repeated measures ANOVAs in Experiment 2 and with synchrony × configuration (upright or inverted) repeated measures ANOVAs in Experiment 3. Brain responses were calculated as in Experiment 1. Note that this means that we now calculated the base rate response as the sum of the first 10 and not the first 8 harmonics because the base rate in Experiments 2–3 no longer overlapped with power-line artifacts at 50 and 100 Hz. Analyses were conducted on the same four electrode clusters as in Experiment 1 (see Supplementary Fig. 2 for collapsed topographies). However, since Experiments 2–3 had only 64 electrodes, instead of the 128 electrodes in Experiment 1, we used 3 instead of 5 electrodes placed around the center electrode of the 4 clusters (i.e., Oz, PO7, PO8, and FCz). The resulting data were analyzed with a synchrony × fluency × region repeated measures ANOVA in Experiment 2 and with a synchrony × configuration × region repeated measures ANOVA in Experiment 3. All other details were as in Experiment 1. See Supplementary Tables 2–3 for means and standard deviations per condition.

### Results

#### Rating Data

**Figure 4:**
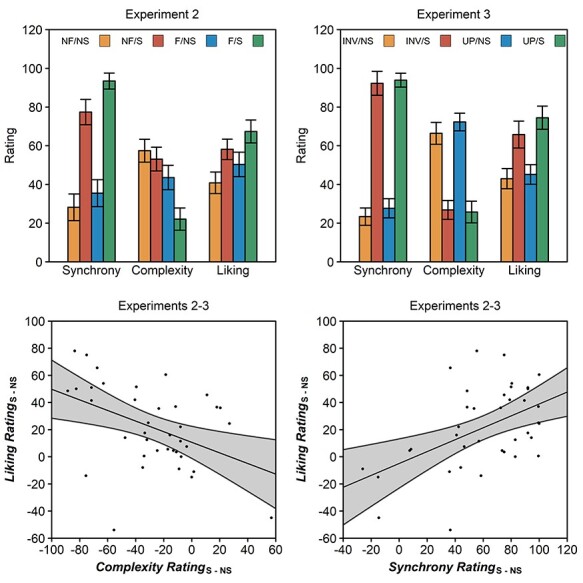
Rating data of Experiments 2 and 3. Top. Synchrony, complexity, and liking ratings of fluent (F), non-fluent (NF), upright (UP), inverted (INV), synchronous (S), or non-synchronous (NS) stimuli in Experiments 2 and 3. Error bars are SEMs corrected for within-subject designs (Morey 2008). Bottom. Scatter plots across Experiments 2 and 3 showing how the difference in perceived complexity (left) and perceived synchrony (right) between synchronous and non-synchronous stimuli correlates with the difference in liking between synchronous and non-synchronous stimuli. The shaded gray area shows the 95% confidence interval of a regression.

The synchrony ratings of Experiment 2 revealed a main effect of synchrony, *F*(1, 18) = 39.46, *P* < 0.001, η*_p_*^2^ = 0.69, with higher synchrony ratings for synchronous than for asynchronous movements, and a main effect of fluency, *F*(1, 18) = 7.24, *P* = 0.015, η*_p_*^2^ = 0.29, with higher synchrony ratings for fluent than for non-fluent movements. None of the other effects were significant. The synchrony ratings of Experiment 3 likewise revealed a main effect of synchrony, *F*(1, 18) = 93.58, *P* < 0.001, η*_p_*^2^ = 0.84, with higher synchrony ratings for synchronous than for asynchronous movements, but no other significant effects (Fig. 4).

The complexity ratings of Experiment 2 revealed a main effect of fluency, *F*(1, 18) = 11.82, *P* = 0.003, η*_p_*^2^ = 0.40, with higher complexity ratings for non-fluent than for fluent movements, and a synchrony × fluency interaction, *F*(1, 18) = 4.84, *P* = 0.041, η*_p_*^2^ = 0.21, with a larger difference between non-fluent and fluent movements in the synchronous than in the asynchronous condition. The complexity ratings of Experiment 3 revealed only a main effect of synchrony, *F*(1, 18) = 36.26, *P* < 0.001, η*_p_*^2^ = 0.67, with higher complexity ratings for asynchronous than for synchronous movements. None of the other effects were significant (Fig. 4).

The liking ratings of Experiment 2 revealed a main effect of synchrony, *F*(1, 18) = 7.82, *P* = 0.012, η*_p_*^2^ = 0.30, with higher liking ratings for synchronous than for asynchronous movements. The same effect of synchrony was also found in Experiment 3, *F*(1, 18) = 11.24, *P* = 0.004, η*_p_*^2^ = 0.38. None of the other effects were significant (Fig. 4).

#### Synchrony and Fluency

**Figure 5:**
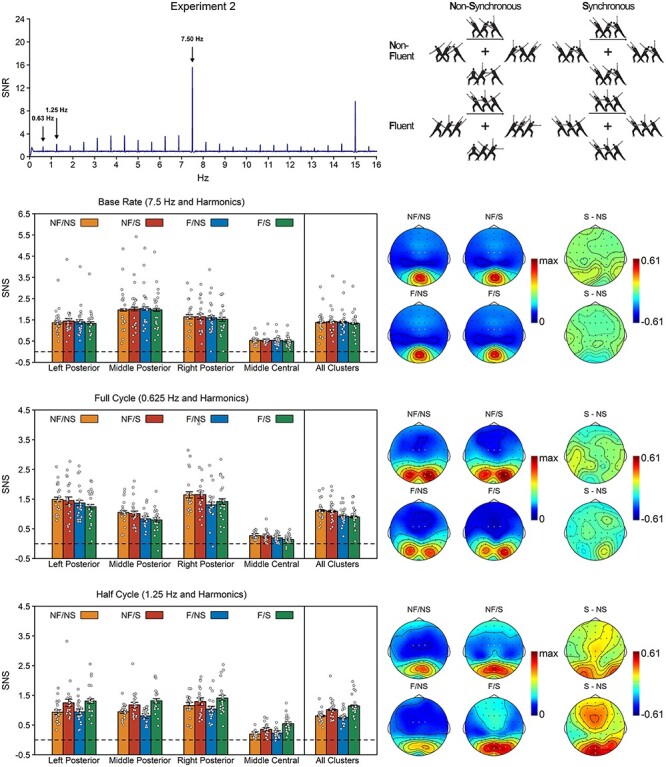
Results of Experiment 2. Top left. SNR across all participants, conditions, and electrodes of interest. Note that Experiment 2 used a different base rate (7.5 Hz) than Experiment 1 (10 Hz). See Supplementary Fig. 4 for plots per condition. Top right. Stimuli of Experiment 2. Participants observed four agents making fluent (F) or non-fluent (NF) movements in synchrony (S) or out of synchrony (NS). For each condition, three consecutive postures are shown per agent. Bottom. SNS per response and condition, together with their topographies. Topographies are plotted for each of the four conditions, together with the topography of the synchrony effect (S–NS) separately for fluent and non-fluent trials. The condition topographies are scaled from 0 to the maximum amplitude across the four conditions. The synchrony effect topographies are scaled symmetrically around zero, with the outer values based on the maximum synchrony effect across the six difference topographies. The white dots on the topographies indicate the electrodes included in the analyses. The white dots on the barplots represent the data of the individual participants. Error bars are SEMs corrected for within-subject designs (Morey 2008).

Experiment 2 tested the hypothesis that synchronous movements are bound into a percept of group movement. If this is the case, we should see stronger half cycle responses in the synchronous than in the asynchronous condition. In addition, Experiment 2 also tested whether binding postures into movements is a prerequisite for binding movements into groups. If so, then the effect of synchrony should depend on the quality of the movement representations and should be modulated by movement fluency. In line with these hypotheses, the half cycle analysis of Experiment 2 revealed a main effect of synchrony, *F*(1, 18) = 33.56, *P* < 0.001, BF_10_ = 1.35 × 10^3^, η*_p_*^2^ = 0.65, with stronger responses for synchronous than for asynchronous movements, and a synchrony × fluency interaction, *F*(1, 18) = 7.36, *P* = 0.014, BF_10_ = 3.88, η*_p_*^2^ = 0.29, with a stronger synchrony effect for fluent, *t*(18) = 6.13, *P* < 0.001, *d*_z_ = 1.41, BF_10_ = 2.5 × 10^3^, than for non-fluent movements, *t*(18) = 3.58, *P* = 0.002, *d*_z_ = 0.82, BF_10_ = 19.18. In line with Experiment 1, the half cycle topography was characterized by a posterior cluster spreading out over both middle and lateral posterior electrodes and an additional weaker cluster over frontocentral electrodes (Fig. 5). This was confirmed by a main effect of region, *F*(3, 54) = 78.56, *P* < 0.001, η*_p_*^2^ = 0.81, which indicated that half cycle responses were significant in all clusters, all *t*(18) > 11.08, *P*_one-tailed_ < 0.001, *d*_z_ ≥ 2.54, BF_10_ ≥ 1.21 × 10^7^, but stronger over posterior clusters than over the frontocentral cluster, all *t*(18) ≥ 11.01, *P* < 0.001, *d*_z_ ≥ 2.53, BF_10_ ≥ 5.56 × 10^6^. None of the other effects reached significance.

The full cycle and base rate analyses revealed a different pattern than the half cycle analysis. The full cycle analysis revealed no main effect of synchrony, *F*(1, 18) = 0.46, *P* = 0.505, BF_10_ = 0.29, η*_p_*^2^ = 0.03, but instead revealed a main effect of fluency, *F*(1, 18) = 49.37, *P* < 0.001, BF_10_ = 1.25 × 10^4^, η*_p_*^2^ = 0.73, with stronger responses for non-fluent than for fluent movements. As in Experiment 1, the full cycle topography was characterized by a left and right posterior cluster (Fig. 5). This was confirmed by a main effect of region, *F*(2.26, 40.66) = 61.53, *P* < 0.001, η*_p_*^2^ = 0.77, which indicated that the full cycle response was stronger over left and right posterior clusters than over the other two clusters, all *t*(18) ≥ 4.45, *P* < 0.001, *d*_z_ ≥ 1.02, BF_10_ ≥ 102.43. None of the other effects reached significance.

The base rate analysis revealed only a main effect of region, *F*(2.06, 37.13) = 30.34, *P* < 0.001, η*_p_*^2^ = 0.63. In line with Experiment 1, this region effect indicated that base rate responses were stronger in the middle posterior cluster than in the other three clusters, all *t*(18) ≥ 2.32, *P* ≤ 0.033, *d*_z_ ≥ 0.53, BF_10_ ≥ 1.99 (Fig. 5). None of the other effects reached significance.

#### Synchrony and Configuration

**Figure 6:**
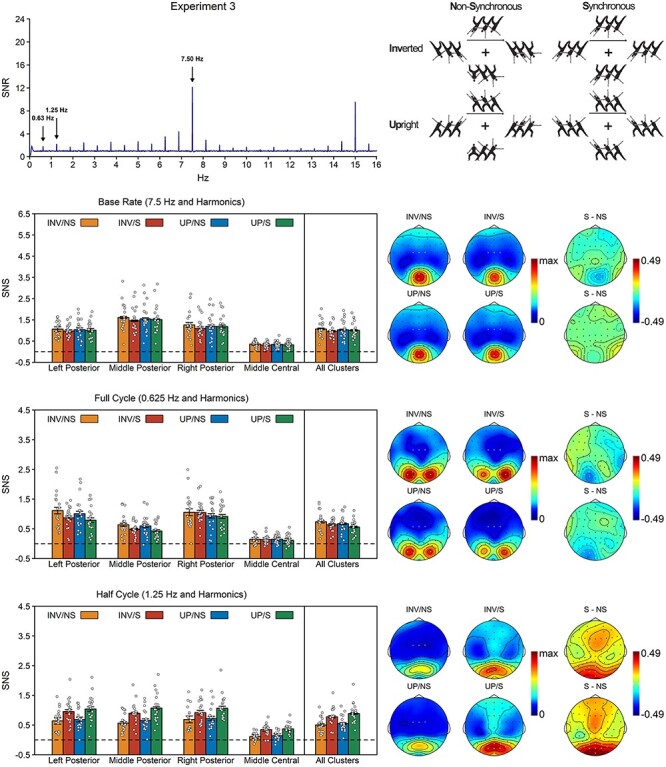
Results of Experiment 3. Top left. SNR across all participants, conditions, and electrodes of interest. Note that Experiment 3 used a different base rate (7.5 Hz) than Experiment 1 (10 Hz). See Supplementary Fig. 5 for plots per condition. Top right. Stimuli of Experiment 3. Participants observed four upright (UP) or inverted (INV) agents moving either in synchrony (S) or out of synchrony (NS). For each condition, three consecutive postures are shown per agent. Bottom. SNS per response and condition, together with their topographies. Topographies are plotted for each of the four conditions, together with the topography of the synchrony effect (S–NS) separately for upright and inverted trials. The condition topographies are scaled from 0 to the maximum amplitude across the four conditions. The synchrony effect topographies are scaled symmetrically around zero, with the outer values based on the maximum synchrony effect across the six difference topographies. The white dots on the topographies indicate the electrodes included in the analyses. The white dots on the barplots represent the data of the individual participants. Error bars are SEMs corrected for within-subject designs (Morey 2008).

Experiment 3 aimed to replicate the effect of synchrony on half cycle responses and additionally tested whether this effect was sensitive to body configuration. If the process of grouping by synchrony is specific to configural shapes, the influence of synchrony on half cycle responses should be stronger for upright than for inverted bodies. If it is not, there should instead be independent effects of synchrony and body configuration. Finally, if our task does not involve any configural processing, there should be no effect of body configuration at all. Supporting independent processes, the half cycle analysis revealed a main effect of synchrony, *F*(1, 18) = 65.77, *P* < 0.001, BF_10_ = 7.76 × 10^4^, η*_p_*^2^ = 0.79, with stronger responses for synchronous than for asynchronous movements, and a main effect of configuration, *F*(1, 18) = 6.97, *P* = 0.017, BF_10_ = 3.41, η*_p_*^2^ = 0.28, with stronger responses for upright than for inverted agents, but no synchrony × configuration interaction, *F*(1, 18) = 1.18, *P* = 0.291, BF_10_ = 0.40, η*_p_*^2^ = 0.06. The half cycle topography was again characterized by a strong posterior and a weaker frontocentral cluster (Fig. 6). This was confirmed by a main effect of region, *F*(2.13, 38.42) = 53.69, *P* < 0.001, η*_p_*^2^ = 0.75, indicating that half cycle responses were significant in all clusters, all *t*(18) > 9.34, *P*_one-tailed_ < 0.001, *d*_z_ ≥ 2.14, BF_10_ ≥ 1.04 × 10^6^, but stronger over posterior clusters than over the frontocentral cluster, all *t*(18) ≥ 8.34, *P* < 0.001, *d*_z_ ≥ 1.91, BF_10_ ≥ 1.12 × 10^5^. None of the other effects reached significance.

The full cycle and base rate analyses again revealed a different pattern than the half cycle analysis. Although the full cycle analysis also indicated a main effect of synchrony, *F*(1, 18) = 9.29, *P* = 0.007, BF_10_ = 7.08, η*_p_*^2^ = 0.34, and a borderline non-significant effect of configuration, *F*(1, 18) = 4.31, *P* = 0.052, BF_10_ = 1.36, η*_p_*^2^ = 0.19, both effects went in the opposite direction as the half cycle results. That is, the full cycle analysis revealed not stronger but weaker responses for synchronous and upright movements. As before, the full cycle topography consisted of a left and right posterior cluster (Fig. 6). This was confirmed by a main effect of region, *F*(2.23, 40.14) = 54.90, *P* < 0.001, η*_p_*^2^ = 0.75, indicating that the full cycle response was stronger over left and right posterior clusters than over the other two clusters, all *t*(18) ≥ 5.99, *P* < 0.001, *d*_z_ ≥ 1.38, BF_10_ ≥ 1.96 × 10^3^. None of the other effects reached significance.

The base rate analysis also revealed a reverse synchrony effect, *F*(1, 18) = 7.02, *P* = 0.016, BF_10_ = 3.48, η*_p_*^2^ = 0.28, with stronger responses for asynchronous movements, and further showed a region × synchrony × configuration interaction, *F*(3, 54) = 4.28, *P* = 0.009, η*_p_*^2^ = 0.19. Post-hoc analyses revealed that this three-way interaction was driven by a significant synchrony × configuration interaction in the right posterior cluster, *F*(1, 18) = 6.51, *P* = 0.020, η*_p_*^2^ = 0.13, but not in the other three clusters, all *F*(1,18) ≤ 3.25, all *P* ≥ 0.088, η*_p_*^2^ ≤ 0.06. This two-way interaction indicated that responses over right posterior electrodes were stronger for asynchronous movements for inverted, *t*(18) = 3.32, *P* = 0.004, BF_10_ = 11.80, *d*_z_ = 0.76, but not for upright stimuli, *t*(18) = 0.04, *P* = 0.969, BF_10_ = 0.24, *d*_z_ = 0.01. In addition, and in line with Experiments 1–2, there was also a main effect of region, *F*(2.16, 38.82) = 48.17, *P* < 0.001, indicating stronger responses in the middle posterior cluster than in the other three clusters, all *t*(18) ≥ 3.73, *P* ≤ 0.002, *d*_z_ ≥ 0.86, BF_10_ ≥ 25.75 (Fig. 6). None of the other effects reached significance.

### Interim Discussion

Experiments 2 and 3 investigated the hypothesis that the brain binds multiple, temporally related movements into a single group movement. Supporting this hypothesis, both experiments showed that half cycle responses were stronger for synchronous than for asynchronous movements. Experiment 2 further showed that this effect was stronger for fluent than for non-fluent sequences, thereby confirming our hypothesis that binding postures into movements (Stage 2) is a prerequisite for binding movements into groups (Stage 3; Fig. 1). Two points merit further discussion, however. First, the effect of synchrony did not disappear entirely for non-fluent sequences. Such residual synchrony effect could be taken as evidence that not only movements but also postures were bound into groups, but this is unlikely considering that posture grouping should primarily affect full cycle responses. Instead, a residual synchrony effect in the non-fluent condition likely indicates that temporal scrambling only partially disrupted the binding of postures into movements (Lange and Lappe 2007). Second, the main effect of fluency, though prominent in Experiment 1, disappeared in Experiment 2. This is likely because synchrony dominated the effect of fluency. Indeed, full cycle responses, which were less sensitive to synchrony, did show a clear fluency effect similar to Experiment 1.

Experiment 3 additionally showed that half cycle responses were sensitive not just to movement synchrony but also to body inversion. This indicates that these responses captured configural movement processing (Grossman and Blake 2001; Orgs et al. 2011), the core mechanism underlying biological motion perception (Vaina et al. 2001; Giese and Poggio 2003; Lange and Lappe 2006). However, while fluency interacted with synchrony (Experiment 2), inversion did not (Experiment 3). This suggests that the process of binding movements into groups is relevant *for* but not specific *to* configural movement processing, similar to recent work showing that successful navigation through a group of people involves independent processing of biological motion and optical flow (Riddell and Lappe 2018; Mayer et al. 2019). More specifically, our results suggest that synchrony is processed independent of configural shape and may instead involve a combination of local and global features.

Finally, Experiments 2–3 also included behavioral judgments. These judgments showed that fluent and synchronous movements were perceived as more synchronous than non-fluent or asynchronous stimuli, whereas non-fluent and asynchronous movements were perceived as more complex than fluent or synchronous movements. In addition, synchronous stimuli were also perceived as more aesthetically pleasing than asynchronous stimuli (see also Eskenazi et al. 2015; Vicary et al. 2017). These data are consistent with the idea that fluently processed stimuli produce a hedonic response (Reber et al. 2004). Indeed, exploratory Spearman correlations across both experiments showed that the difference in liking between synchronous and asynchronous stimuli correlated positively with the difference in perceived synchrony, ρ = 0.44, *P* = 0.006, but negatively with the difference in perceived complexity, ρ = −0.42, *P* = 0.008 (Fig. 4).

## Dissociating Full and Half Cycle Responses

All three experiments suggest a double dissociation of half and full cycle frequencies. Half cycle frequencies increased with the saliency of apparent movement but decreased with the saliency of static body postures; full cycle frequencies instead increased with the saliency of static body postures but decreased with the saliency of apparent movement. However, given that the half cycle response was a harmonic of the full cycle response, it is important to also demonstrate this double dissociation formally. Therefore, to directly test the hypothesis that full and half cycle responses were inversely influenced by our manipulations, we investigated how amplitudes at these responses in the left and right posterior electrode clusters (i.e., the only two clusters that were consistently activated for both responses) were influenced by movement fluency (Experiments 1 and 2), synchrony (Experiments 2 and 3), and body configuration (Experiment 3).

The results of a frequency × fluency × region repeated measures ANOVA on the data of Experiment 1 revealed only a frequency × fluency interaction, *F*(1, 9) = 37.05, *P* < 0.001, BF_10_ = 171.43, η*_p_*^2^ = 0.80, indicating that the full cycle response decreased, *t*(9) = −4.13, *p* = 0.003, BF_10_ = 18.77, *d*_z_ = 1.30, but the half cycle response increased, *t*(9) = 6.85, *P* < 0.001, BF_10_ = 366.53, *d*_z_ = 2.17, on fluent compared with non-fluent trials.

A frequency × synchrony × fluency × region repeated measures ANOVA on the data of Experiment 2 likewise revealed a frequency × fluency interaction, *F*(1, 18) = 10.42, *P* = 0.005, BF_10_ = 9.87, η*_p_*^2^ = 0.37, with smaller full cycle responses, *t*(18) = −6.80, *P* < 0.001, BF_10_ = 8.4 × 10^3^, *d*_z_ = 1.56, but not half cycle responses, *t*(18) = 0.27, *P* = 0.787, BF_10_ = 0.25, *d*_z_ = 0.06, on fluent compared with non-fluent trials, as well as a frequency × synchrony interaction, *F*(1, 18) = 21.50, *P* < 0.001, BF_10_ = 148.23, η*_p_*^2^ = 0.54, with larger half cycle responses, *t*(18) = 4.56, *P* < 0.001, BF_10_ = 128.17, *d_z_* = 1.05, but not full cycle responses, *t*(18) = −0.23, *P* = 0.818, BF_10_ = 0.24, *d*_z_ = 0.05, on synchronous compared with asynchronous trials.

Finally, a frequency × synchrony × configuration × region repeated measures ANOVA on the data of Experiment 3 revealed a frequency × synchrony interaction, *F*(1, 18) = 34.46, *P* < 0.001, BF_10_ = 1.56 × 10^3^, η*_p_*^2^ = 0.66, with increased half cycle responses, *t*(18) = 8.02, *P* < 0.001, BF_10_ = 6.72 × 10^4^, *d*_z_ = 1.84, but reduced full cycle responses, *t*(18) = −2.34, *P* = 0.031, BF_10_ = 2.08, *d*_z_ = 0.54, on synchronous compared with asynchronous trials, and a frequency × configuration interaction, *F*(1, 18) = 15.70, *P* = 0.001, BF_10_ = 39.98, η*_p_*^2^ = 0.47, with larger half cycle responses, *t*(18) = 2.12, *P* = 0.049, BF_10_ = 1.45, *d*_z_ = 0.49, but weaker full cycle responses, *t*(18) = −2.41, *P* = 0.027, BF_10_ = 2.31, *d*_z_ = 0.55, for upright compared with inverted stimuli.

Taken together, these results indicate that manipulations that tended to strengthen the half cycle response also tended to weaken the full cycle response. This is consistent with the hypothesis that these two responses captured dissociable processes, despite being harmonically related. Specifically, our results suggest that full cycle responses captured the repetition of arbitrary posture sequences, whereas half cycle responses captured the completion of a body movement (Fig. 1).

## General Discussion

This study tested the hypothesis that group movement is processed in the motion-from-structure pathway along three hierarchical stages (Fig. 1). First, body postures are processed individually (Stage 1); next, successive body postures are bound into a continuous movement percept (Stage 2); finally, temporally related movements are integrated into holistic group representations (Stage 3). To test this hypothesis, we developed a new EEG frequency tagging paradigm that elicited responses at three distinct frequencies: at half cycle, tagging movement completion (Stage 2), at full cycle, tagging the repetition of posture sequences (Stage 1), and at base rate, tagging image presentation (Stage 1). Across three experiments, we then tested how these responses were modulated by fluency, inversion, and synchrony.

Half cycle responses were stronger for fluent than for non-fluent sequences (Experiment 1) and for upright than for inverted bodies (Experiment 3). Both temporal scrambling (Orgs et al. 2013, 2016) and body inversion (Orgs et al. 2011) are well-known to perturb configural movement processing. Especially inversion is considered a hallmark of configural movement perception because it perturbs global shape processing while leaving local features intact. By doing so, inversion interferes specifically with the core process underlying biological motion perception: the integration of movement with global form (Giese and Poggio 2003; Lange and Lappe 2006). Hence, the inversion effect on half cycle responses supports the hypothesis that these responses captured processes relevant to biological motion perception.

Experiments 2–3 further showed that half cycle responses were stronger for synchronous than for asynchronous movements. This effect was stronger for fluent than for non-fluent sequences but did not depend on body configuration. This indicates that synchronous movements are integrated into a single group movement after the temporal integration of postures into movements (Giese and Poggio 2003; Lange and Lappe 2006), so that rather than having to analyze the movements of all individual actors, a more efficient movement analysis can take place at the group level. While this process of binding movements into groups did not appear specific to configural movement perception (i.e., no synchrony × configuration interaction), it was clearly relevant for it, as the half cycle response it influenced did show specificity to configural shapes (i.e., main effect of configuration).

Importantly, the finding that half cycle responses captured processes relevant to biological motion processing should not be taken to imply that these responses are unique to the human form. Although biological motion perception typically involves human shapes, perceptual learning studies have shown that the same mechanisms are also used to process movements made by complex, artificial stimuli (Jastorff et al. 2006, 2009). For this reason, we explicitly decided against using control conditions with degraded body stimuli or simple shapes (Orgs et al. 2011, 2016). Such stimuli also inevitably differ in terms of low-level visual features. Significant differences between body stimuli and other inanimate moving objects could therefore always be attributed to low-level differences between their apparent motion paths. Indeed, it has previously been shown that body inversion produces smaller effect sizes than body scrambling, arguably because inverted body stimuli are better matched for low-level visual features (Orgs et al. 2011). This suggests that body inversion, by eliminating global but not local shape cues, is the strongest possible test for configural form constraints on motion perception, the core mechanism underlying biological motion processing (Vaina et al. 2001; Giese and Poggio 2003).

Opposite to half cycle responses, full cycle responses were reduced when seeing synchronous, fluent, or upright movement. This suggests that perturbing movement processing caused frame-by-frame processing to take over. The finding that full cycle responses were, if anything, stronger for inverted relative to upright bodies further indicates that this frame-by-frame processing was not a configural process (Reed et al. 2003; Lange and Lappe 2006, 2007), but reflected a more local posture analysis. Such an interpretation is consistent with the lateral-occipital topography of the full cycle response, speculatively pointing to an involvement of the extra-striate visual cortex, including extra-striate body area (EBA). Indeed, brain imaging (Brandman and Yovel 2010) and brain stimulation (Urgesi et al. 2007) studies have shown that EBA is equally or even more sensitive to inverted than to upright bodies.

Similar to full cycle responses, base rate responses were either not influenced by synchrony (Experiment 2) or were stronger for asynchronous movements (Experiment 3). In line with the full cycle results, this suggests that perturbing movement perception triggers static frame-by-frame processing. This result is seemingly inconsistent with the results of a recent similar study in which frequency tagging was used to measure brain responses to periodic contrast changes of four point-light dancers moving in or out of synchrony (Alp et al. 2017). Specifically, this study found increased responses to contrast changes in the synchrony condition. As these contrast changes were independent of the dancers’ movements, the responses measured by Alp et al. (2017) did not reflect movement processing per se, but rather the processing of stimulus features in a context of movement, similar to the base rate here. Yet, in contrast to our base rate results, Alp et al. (2017) found that posterior, occipital areas responded more strongly, rather than less, to contrast changes when dancers moved synchronously. Their study used two frequencies, however, with contrast changing at F1 for one half of the dancers and at F2 for the other half. This generates not only fundamental responses at the stimulation frequencies but also intermodulation responses at linear combinations of those frequencies (e.g., F1 + F2). These intermodulation responses reflect non-linear interactions between the two input streams (Zemon and Ratcliff 1984; Norcia et al. 2015). In Alp et al. (2017), synchrony modulated the intermodulation but not the stimulation frequencies, suggesting that synchrony influenced not stimulus processing per se, but rather the integration of visual features across stimuli. These differences in design might explain why we found reduced rather than increased base rate responses in the synchronous condition.

More generally, a key difference between our study and Alp et al. (2017) is that in addition to measuring static visual features in a context of movement, we also measured different components of the movement itself. However, in apparent motion paradigms, it is not possible to tag these components at fully independent frequencies (e.g., as in Liu-Shuang et al. 2014). Nevertheless, half and full cycle responses were influenced in opposite ways by our manipulations and had different topographies. This suggests that, even though they were harmonically related, half and full cycle frequencies captured distinct processes. These results are consistent with frequency tagging studies in the auditory domain, showing that different components of a musical rhythm such as beat and meter (Nozaradan et al. 2011), beat and rhythmic tapping (Nozaradan et al. 2015), or different meters (Chemin et al. 2014) can likewise reflect distinct levels of rhythm perception, despite being harmonically related.

Finally, by investigating how synchrony interacts with movement perception, the current study has important implications for understanding higher-order social phenomena such as group alignment (Raafat et al. 2009; Shamay-Tsoory et al. 2019) and its social consequences (Rennung and Göritz 2016). In particular, our results show that collective group movement forms a strong visual trigger to which the brain responds more strongly than to multiple agents moving individually. Such enhanced neural responding to collective behavior may in turn play a critical role in the tendency of humans (Dyer et al. 2009; Raafat et al. 2009) and animals (Sumpter 2006; Couzin 2018) to align their behavior with that of the group. Similarly, the same mechanism may also contribute to the fact that people find synchrony aesthetically pleasing (Eskenazi et al. 2015; Vicary et al. 2017) and see it as a signal of group cohesion (Lakens and Stel 2011; Marques-Quinteiro et al. 2019; Wilson and Gos 2019), as stimuli that are processed more fluently are known to produce a hedonic response (Reber et al. 2004).

Our study further adds onto a growing body of research investigating ensemble processes in social perception. This research has shown that two individuals are represented as a single unit when they are facing (Papeo et al. 2017; but see also Vestner et al. 2020) or interacting (Ding et al. 2017; Liu et al. 2018), and that this changes both how these stimuli are perceived (Papeo et al. 2017; Liu et al. 2018; Vestner et al. 2019) and remembered (Ding et al. 2017; Vestner et al. 2019). By showing that, despite identical input, the brain responds more strongly to people moving together than to people moving independently, the current study reveals a potential neural correlate of how ensembles shape social perception and evaluation.

To conclude, the current research makes two important contributions. First, it introduces a new EEG paradigm that can simultaneously capture static and dynamic processing stages of configural movement perception at different frequencies of stimulus presentation. Second, it extends existing models of biological motion perception by showing that the brain not only binds bodies into movements (Giese and Poggio 2003; Lange and Lappe 2006) but also movements into groups. Enhanced perceptual processing of movement synchrony may form the basis for higher-level social phenomena such as group alignment and its social consequences.

## Supplementary Material

SyncPaper_CerCor_SupMat_bhab385

Exp1_F_bhab385

Exp1_NF_bhab385

Exp1_RND_bhab385

Vids_Exp2_bhab385

Vids_Exp3_bhab385
