# Supplementary material for: EEG Frequency Tagging Reveals the Integration of Form and Motion Cues into the Perception of Group Movement
Source: Cereb Cortex. 2021 Nov 4;32(13):2843–57. doi: 10.1093/cercor/bhab385 (PMC9247417; doi:10.1093/cercor/bhab385)
Supplement: SyncPaper_CerCor_SupMat_bhab385 [file syncpaper_cercor_supmat_bhab385.pdf]

Supplementary Material

Means and Standard Deviations

Table S1. *M* ± *SD* of the SNS amplitudes across ROIs in Experiment 1

	Random	Non-Fluent	Fluent
Base Rate	1.34 ± 0.33	1.54 ± 0.36	1.16 ± 0.35
Full Cycle	0.02 ± 0.12	1.52 ± 0.54	0.88 ± 0.22
Half Cycle	-0.03 ± 0.05	1.24 ± 0.22	2.28 ± 0.48

Table S2. *M* ± *SD* of the SNS amplitudes across ROIs in Experiment 2

	Non-Fluent Asynchrony	Non-Fluent Synchrony	Fluent Asynchrony	Fluent Synchrony
Base Rate	1.38 ± 0.64	1.41 ± 0.67	1.39 ± 0.69	1.33 ± 0.61
Full Cycle	1.11 ± 0.38	1.09 ± 0.41	0.93 ± 0.32	0.90 ± 0.40
Half Cycle	0.81 ± 0.22	1.02 ± 0.37	0.75 ± 0.23	1.15 ± 0.42

Table S3. *M* ± *SD* of the SNS amplitudes across ROIs in Experiment 3

	Inverted Asynchrony	Inverted Synchrony	Upright Asynchrony	Upright Synchrony
Base Rate	1.08 ± 0.41	0.96 ± 0.33	1.03 ± 0.46	1.01 ± 0.40
Full Cycle	0.74 ± 0.35	0.64 ± 0.23	0.67 ± 0.30	0.56 ± 0.25
Half Cycle	0.50 ± 0.27	0.78 ± 0.31	0.55 ± 0.26	0.89 ± 0.34

Collapsed Topographies

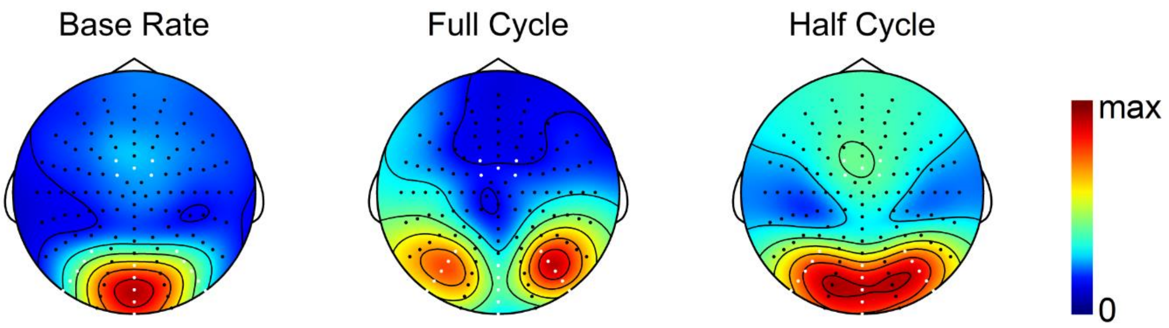

Figure S1. Averaged Topographies Experiment 1. Topographies averaged across all three conditions of Experiment 1. Electrodes included in the analysis were chosen based on these topographies, following the collapsed localizer approach outlined by (Luck and Gaspelin 2017). The electrodes included in the analysis are highlighted using white dots.

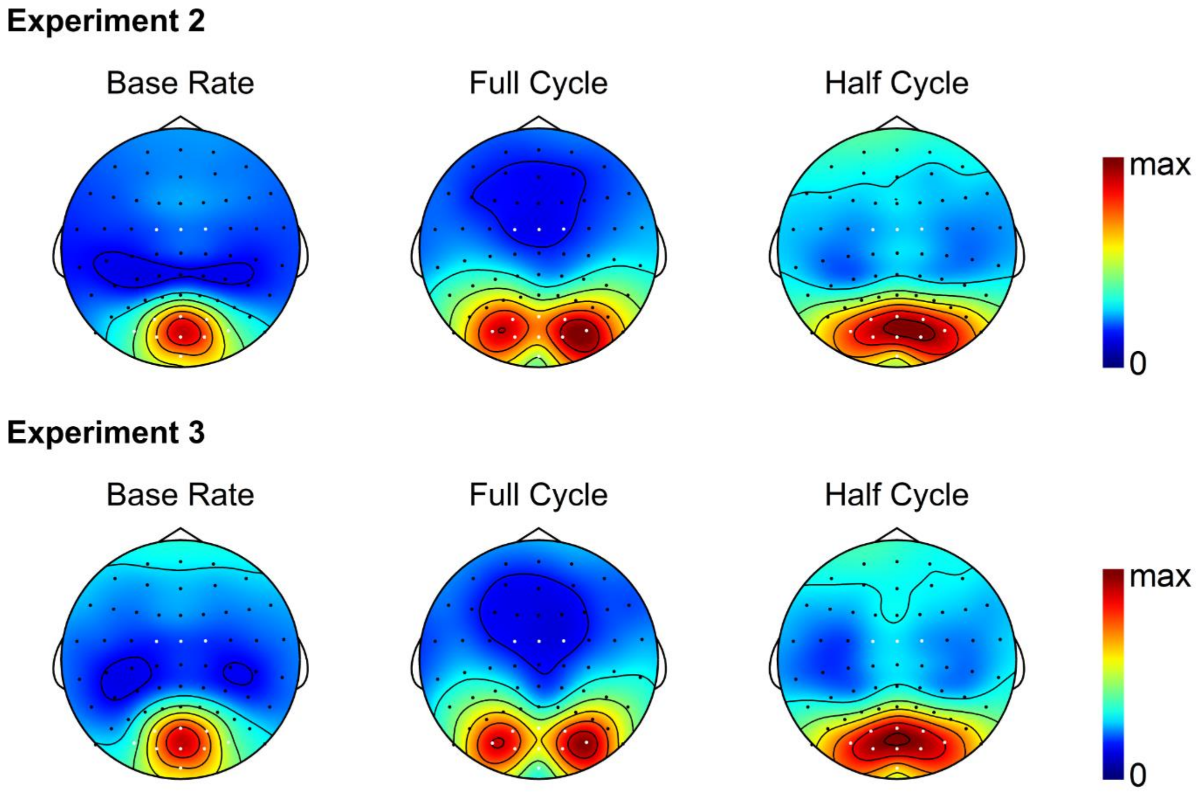

Figure S2. Averaged Topographies Experiments 2-3. Topographies are averaged across all four conditions of Experiments 2 and 3. Electrodes included in the analysis were chosen

based on Experiment 1, but also adequately capture the clusters in the collapsed topography of Experiments 2-3, with the exception of the frontocentral cluster, which is missing from the collapsed topography of Experiments 2-3. The frontocentral cluster was nonetheless included because it was clearly present in Experiment 1 and because collapsed localizers can be insensitive to clusters that are only present in some cells of the design, as was the case here.

Amplitude Spectra Per Condition

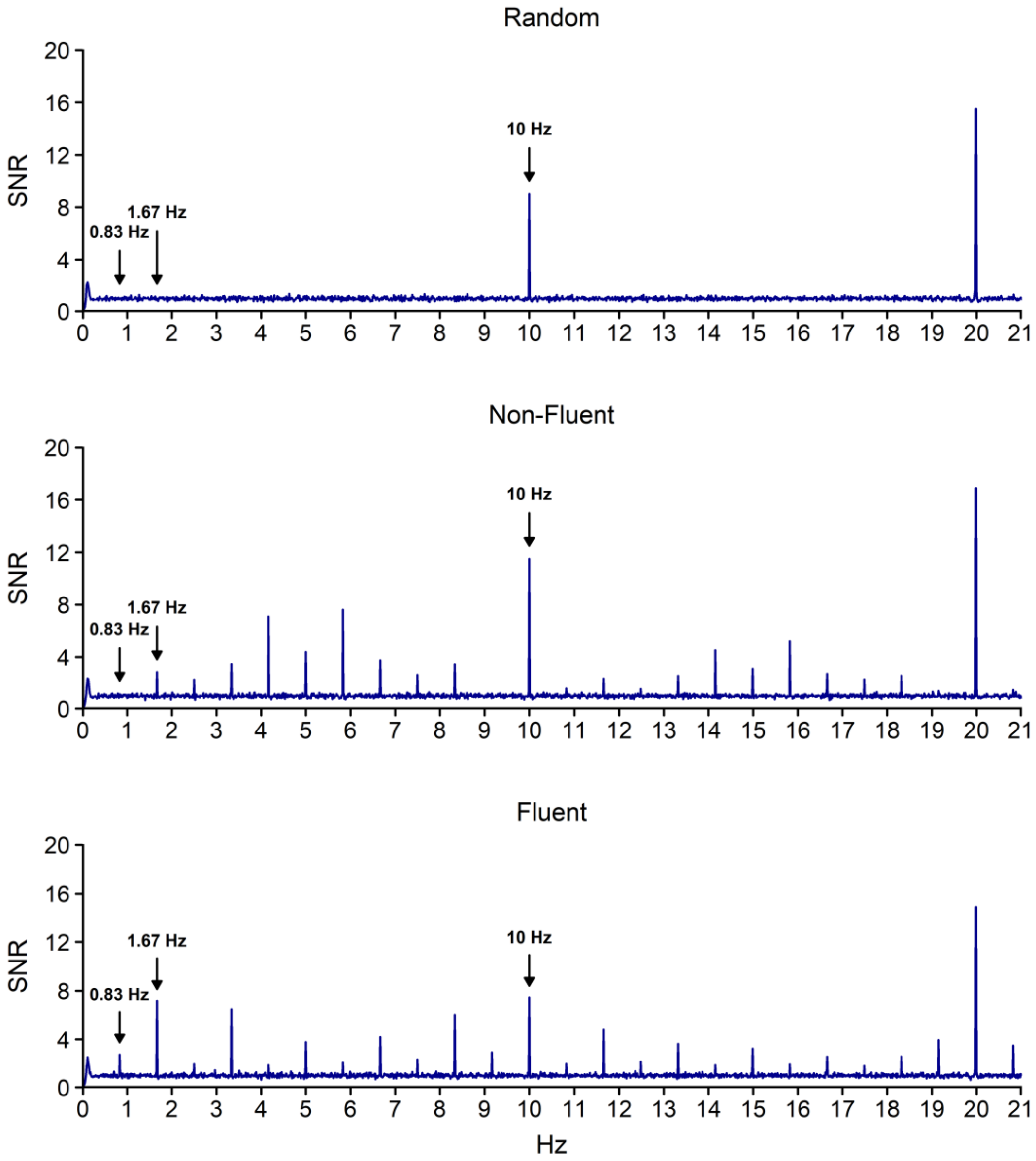

Figure S3. SNR-corrected amplitude spectrum of Experiment 1. The SNR is shown across all participants and electrodes of interest, but separately per condition. Note that the brain response is known to be distributed across harmonics and that how it is distributed depends on the studied process and stimulation procedure (Retter and Rossion 2016). Therefore, the signal at individual frequencies is not necessarily meaningful and summation is necessary.

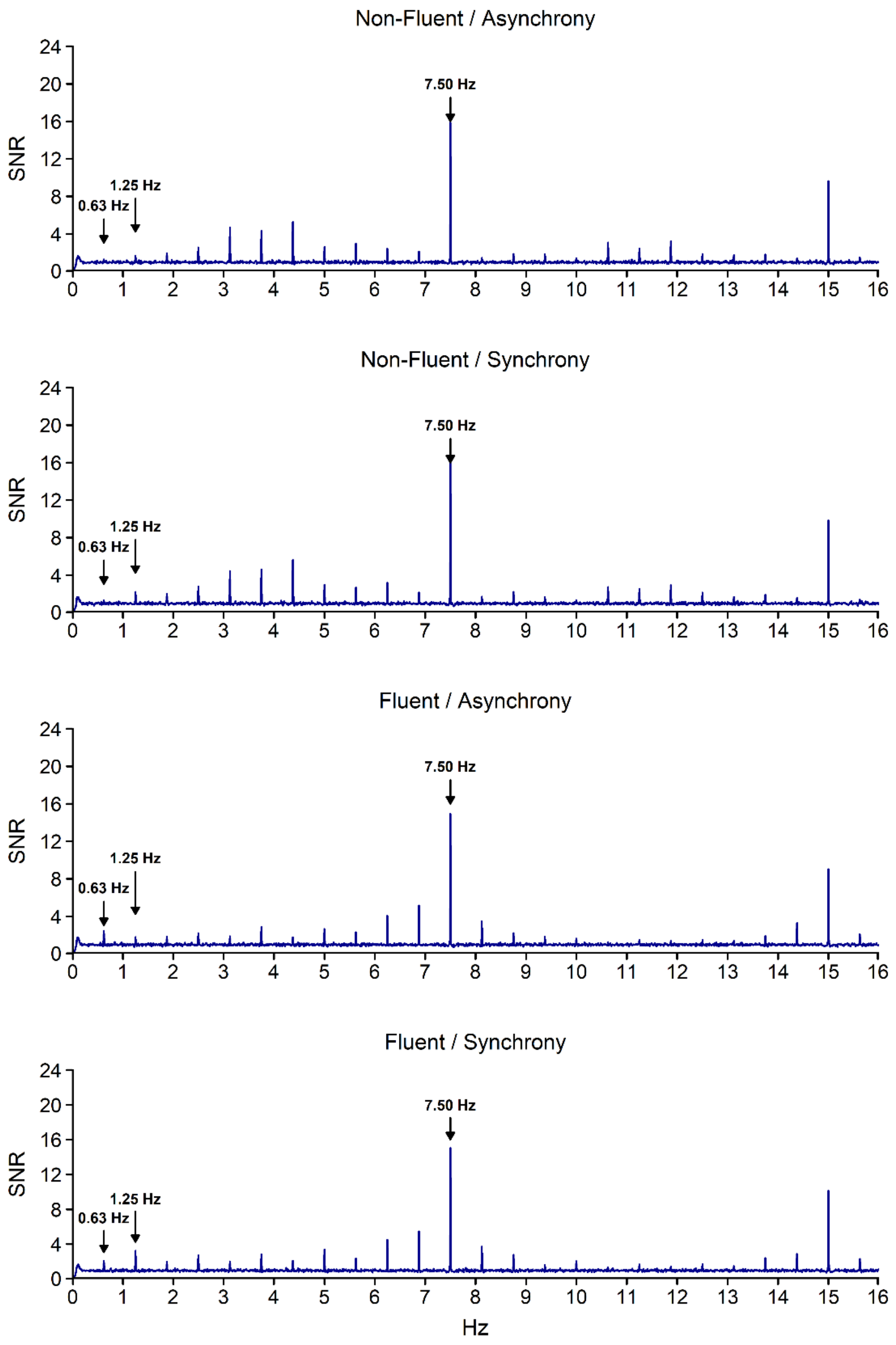

Figure S4. SNR-corrected amplitude spectrum of Experiment 2. The SNR is shown across all participants and electrodes of interest, but separately per condition. Note that the brain response is known to be distributed across harmonics and that how it is distributed depends on the studied process and stimulation procedure (Retter and Rossion 2016). Therefore, the signal at individual frequencies is not necessarily meaningful and summation is necessary.

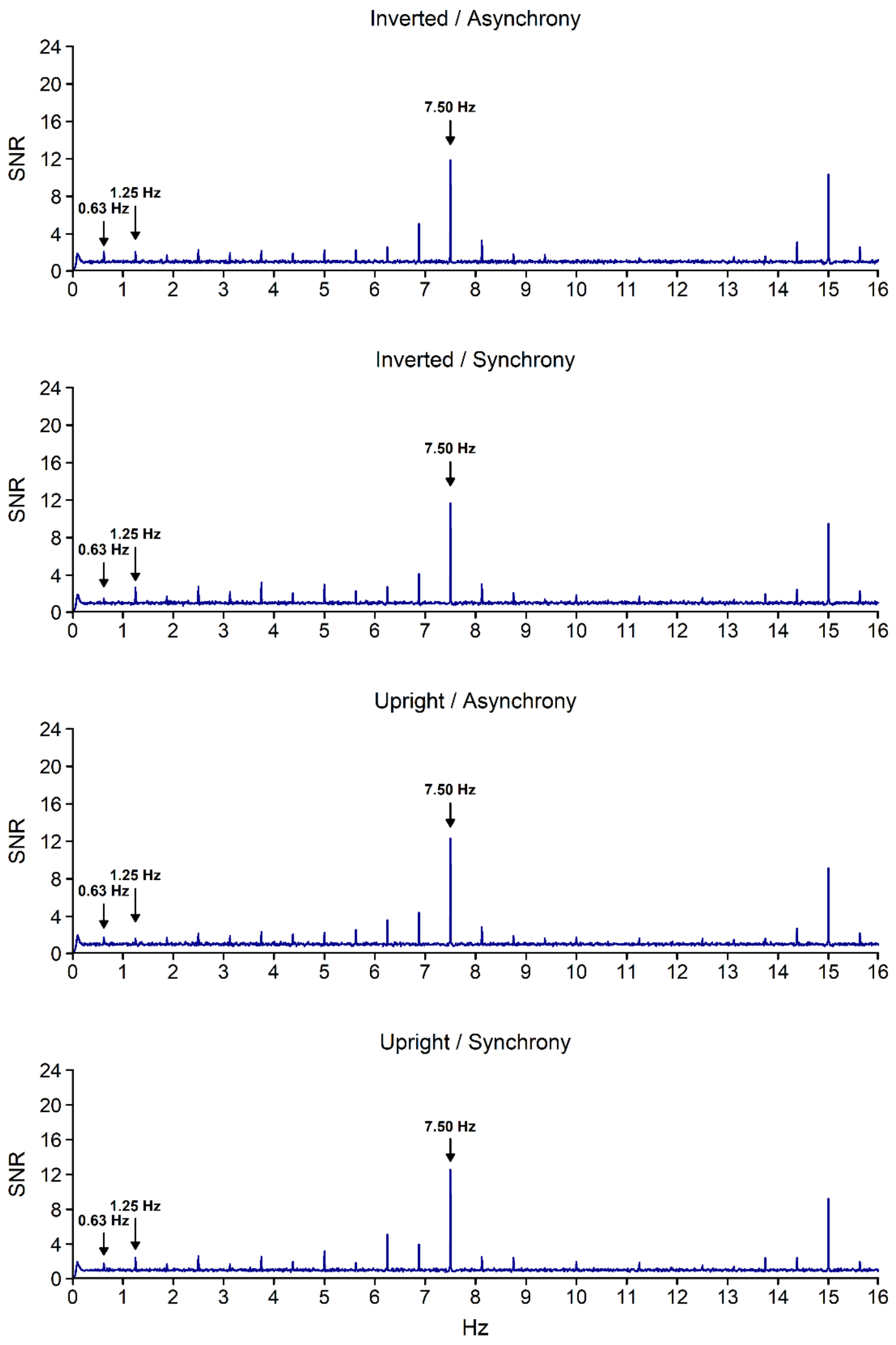

Figure S5. SNR-corrected amplitude spectrum of Experiment 2. The SNR is shown across all participants and electrodes of interest, but separately per condition. Note that the brain response is known to be distributed across harmonics and that how it is distributed depends on the studied process and stimulation procedure (Retter and Rossion 2016). Therefore, the signal at individual frequencies is not necessarily meaningful and summation is necessary.

Supplementary Videos

Video S1. Video showing the stimulus used for the fluent condition in Experiment 1.

Video S2. Video showing the stimulus used for the non-fluent condition in Experiment 1.

Video S3. Video showing the stimulus used for the random condition in Experiment 1.

Video S4. Videos showing the stimuli used in Experiment 2.

Video S5. Videos showing the stimuli used in Experiment 3.
